# The association of fatty liver index and BARD score with all-cause and cause-specific mortality in patients with type 2 diabetes mellitus: a nationwide population-based study

**DOI:** 10.1186/s12933-022-01691-6

**Published:** 2022-12-06

**Authors:** Goh Eun Chung, Su-Min Jeong, Eun Ju Cho, Ji Won Yoon, Jeong-Ju Yoo, Yuri Cho, Kyu-na Lee, Dong Wook Shin, Yoon Jun Kim, Jung-Hwan Yoon, Kyungdo Han, Su Jong Yu

**Affiliations:** 1 Department of Internal Medicine and Healthcare Research Institute, Seoul National University Hospital Healthcare System Gangnam Center, Seoul, Republic of Korea; 2 Department of Family Medicine/Supportive Care Center, Samsung Medical Center, Sungkyunkwan University School of Medicine, Seoul, Republic of Korea; 3 Department of Medicine, Seoul National University College of Medicine, Seoul, Republic of Korea; 4 Department of Internal Medicine and Liver Research Institute, Seoul National University College of Medicine, 101 Daehak-no, Jongno-Gu, Seoul, 03080 Republic of Korea; 5 Department of Internal Medicine, Division of Gastroenterology and Hepatology, Soonchunhyang University Bucheon Hospital, Bucheon, Gyeonggi-do Republic of Korea; 6 Center for Liver and Pancreatobiliary Cancer, National Cancer Center, Goyang, Republic of Korea; 7 Department of Biomedicine & Health Science, Catholic University of Korea, Seoul, Republic of Korea; 8 Department of Clinical Research Design and Evaluation/Department of Digital Health, Samsung Advanced Institute for Health Science, Seoul, Republic of Korea; 9 Department of Statistics and Actuarial Science, Soongsil University, 369 Sangdo-Ro, Dongjak-Gu, Seoul, 06978 Republic of Korea

**Keywords:** Diabetes, Mortality, Cause, Steatosis

## Abstract

**Background:**

Type 2 diabetes and non-alcoholic fatty liver disease (NAFLD) commonly coexist. However, NAFLD’s effect on mortality in Asian patients with type 2 diabetes awaits full elucidation. Therefore, we examined NAFLD-related all-cause and cause-specific mortality in a nationwide Asian population with type 2 diabetes.

**Methods:**

We included patients who had undergone general health checkups between 2009 and 2012 using the National Health Insurance Service database linked to death-certificate data. Hepatic steatosis was defined as a fatty liver index (FLI) ≥ 60, and advanced hepatic fibrosis was determined using the BARD score.

**Findings:**

During the follow-up period of 8.1 years, 222,242 deaths occurred, with a mortality rate of 14.3/1000 person-years. An FLI ≥ 60 was significantly associated with increased risks of all-cause and cause-specific mortality including cardiovascular disease (CVD)-, cancer-, and liver disease (FLI ≥ 60: hazard ratio [HR] = 1.02, 95% confidence interval [CI] 1.01–1.03 for all-cause; 1.07, 1.04–1.10 for CVD; 1.12, 1.09–1.14 for cancer; and 2.63, 2.50–2.77 for liver disease). Those with an FLI ≥ 60 and fibrosis (BARD ≥ 2) exhibited increased risks of all-cause (HR, 95% CI 1.11, 1.10–1.12), CVD- (HR, 95% CI 1.11, 1.09–1.14), cancer- (HR, 95% CI 1.17, 1.15–1.19), and liver disease-related (HR, 95% CI 2.38, 2.29–2.49) mortality.

**Conclusion:**

Hepatic steatosis and advanced fibrosis were significantly associated with risks of overall and cause-specific mortality in patients with type 2 diabetes. Our results provide evidence that determining the presence of hepatic steatosis and/or fibrosis potentially plays a role in risk stratification of mortality outcomes in patients with type 2 diabetes mellitus.

**Supplementary Information:**

The online version contains supplementary material available at 10.1186/s12933-022-01691-6.

## Introduction

Type 2 diabetes mellitus is prevalent worldwide, and health-related burden has increased over the last few decades [1]. As insulin resistance and obesity are common pathogenic factors for type 2 diabetes mellitus and non-alcoholic fatty liver disease (NAFLD), these two diseases commonly coexist, exhibiting a strong relationship [2, 3]. The prevalence of NAFLD in patients with type 2 diabetes mellitus is estimated to be up to 75%, which is more than twice that in the general population [4, 5]. NAFLD and type 2 diabetes mellitus not only coexist but may act synergistically to induce related adverse outcomes due to their shared metabolic risk factors [6–8]. Metabolic syndrome, NAFLD, and imaging biomarkers predicted long-term risk of cardiac events [9]. Subjects with genetic NAFLD and without metabolic disturbances do not have increased cardiovascular risk, whereas those with metabolic disease such as diabetes, have high cardiometabolic risks [10].

The presence of type 2 diabetes mellitus potentially accelerates the risk of advanced hepatic fibrosis [11]. However, the association of NAFLD and fibrosis with the risk of clinical outcomes in specific causes of death among Asian patients with type 2 diabetes mellitus remains unclear. Diabetes in Asian is characterized by early β-cell dysfunction and develops at a younger age, requiring early insulin treatment and posing a higher risk of cardiovascular complications than that in Westerners [12]. Therefore, elucidating the effects of NAFLD and fibrosis on mortality-related outcomes in Asian patients with type 2 diabetes mellitus is of paramount importance.

Although there is controversy regarding routine screening for NAFLD in patients with type 2 diabetes mellitus [13], a previous study reported the cost-effectiveness of NAFLD screening in patients with type 2 diabetes mellitus using ultrasonography plus liver enzymes followed by transient elastography [14]. NAFLD assessed by computed tomography could be an useful tool for identifying type 2 diabetes mellitus patients at higher risk of cardiovascular events [15]. However, imaging modalities have limitations due to technological difficulties, a relatively high cost, and unsuitability for population-based mass screening. Thus, noninvasive biomarkers have been used to predict hepatic steatosis and fibrosis in large populations [16]. The fatty liver index (FLI) and BARD score are easily applicable, as each individual component is a common measurement in clinical practice with acceptable performance in the general population [17–19] and in patients with type 2 diabetes mellitus [20, 21]. In this study, we aimed to investigate the association of the FLI and BARD score with all-cause and cause-specific mortality in patients with type 2 diabetes mellitus based on a population-based, nationwide Korean cohort.

## Methods

### Data source and study setting

This retrospective population-based study was based on the National Health Insurance Service (NHIS) in Korea. Approximately 97% of the Korean population are subscribers to the NHIS and the remaining 3% are receiving medical aid program. The NHIS database contains information about claims submitted by the health care providers for reimbursement, including demographics, medical treatments and procedures, and disease diagnoses based on the International Classification of Diseases, 10th revision (ICD-10). The National Health Screening Program (NHSP) is offered to all insured persons every two years. The NHSP includes self-reported questionnaires for lifestyle behaviors, anthropometric data, and laboratory tests [22].

### Study population

Patients with type 2 diabetes mellitus were defined as follows i) at least one claim per year for the prescription of an antidiabetic medication under ICD-10 codes E11–14 in the insurance claims data or ii) fasting plasma glucose level ≥ 126 mg/dL without insulin and/or a prescription of at least one oral hypoglycemic agent (OHA) [23]. OHAs include sulfonylurea, metformin, meglitinide, a dipeptidyl peptidase-4 inhibitor, an α-glucosidase inhibitor and thiazolidinedione [24].

Among a total of 2,745,689 patients with type 2 diabetes mellitus (age 20 years and older) who participated in health screening between 2009 and 2012, people who met the following criteria were excluded from the study: previous diagnosis of liver cirrhosis (K74, n = 19,645), hepatitis (B15–B19, n = 417,798) before the index year, heavy alcohol consumption (≥ 30 g for men and ≥ 20 g for women of alcohol/day, n = 188,879), or missing information (n = 93,978). Since we confirmed the outcome events after a delay of one year, those with outcome events within one year were excluded (n = 17,614). Finally, 2,007,775 patients with type 2 diabetes mellitus were included in this study. They were followed up to 31 December, one year after the NHSP day, or the date of death, whichever occurred first.

This study was performed in accordance with the ethical guidelines of the 1975 Declaration of Helsinki and approved by the Institutional Review Board of Soongsil University approved this study (SSU-202007-HR-236-01). The requirement for written informed consent was waived because anonymized and de-identified data were used.

### Calculation of the FLI and determination of advanced hepatic fibrosis

We used the FLI to predict fatty liver based on the following components: triglyceride (TG), body mass index (BMI), gamma-glutamyl transferase (GGT) and waist circumference (WC).

The FLI was calculated using the following formula:

$$FLI = \left[ {e^{{(0.953}{ \times \, \ln \left( {TG} \right) \, + \, 0.139 \, \times \, BMI \, + \, 0.718 \, \times \, \ln \left( {GGT} \right) \, + \, 0.053 \, \times \, WC}} -^{15.745)} } \right]/\left[ {1 + e^{{\left( {0.953 \, \times \, \ln \left( {TG} \right) \, + \, 0.139 \, \times \, BMI \, + \, 0.718 \, \times \, \ln \left( {GGT} \right) \, + \, 0.053 \, \times \, WC \, - \, 15.745} \right)}} } \right] \times \, 100$$

A previous study suggested that an FLI score < 30 rules out fatty liver, while that ≥ 60 corresponds to fatty liver with favorable accuracy of diagnosis [25]. In this study, the participants were categorized into three FLI-based groups (< 30 [reference], 30–59, and ≥ 60).

Among the patients with type 2 diabetes mellitus with an FLI ≥ 60, advanced hepatic fibrosis was determined using the BARD score, which is derived from summating the following points: aspartate aminotransferase (AST)/ alanine transaminase (ALT) ratio ≥ 0.8 (two points), BMI ≥ 28 kg/m^2^ (one point), and type 2 diabetes mellitus (one point). A total score of 2 to 4 indicates advanced hepatic fibrosis [19].

### Outcome

The NHIS database was linked to the death certificates from Statistics Korea regarding cause of death and date. Using Korean Standard Classification of Diseases, the cause of death was identified based on ICD-10 codes and specific causes of death were classified as cardiovascular disease (CVD, I00-I99), cancer (including hepatocellular carcinoma, C00-C97), respiratory disease (J00-J99), and liver disease (excluding hepatocellular carcinoma, K70-76) [26].

### Covariates

During the health examination, self-reported, standardized questionnaires were administered to obtain data including alcohol consumption habits, smoking status, and physical activity. Alcohol consumption was classified as non- or mild-to-moderate drinker (< 30 g for men and < 20 g for women of alcohol per day). Smoking status was classified as nonsmoker, former smoker, or current smoker. Regular physical activity was defined as high-intensity exercise 3 or more times a week or moderate-intensity exercise 5 or more times a week. The lowest 20% income proportion was dichotomized into low-income status.

Comorbidities were defined according to the data from the NHSP and each ICD-10 code with a prescription history of related medication. For example, hypertension was defined using ICD-10 codes (I10–13 and I15) with antihypertensive medications, a systolic blood pressure ≥ 140 mmHg, or a diastolic blood pressure ≥ 90 mmHg. Dyslipidemia was defined by ICD-10 code (E78), lipid-lowering medications, or a total cholesterol level greater than 240 mg/dL. The Charlson comorbidity index (CCI) was determined using ICD-10 codes [27]. The severity of type 2 diabetes mellitus was assessed based on the presence of type 2 diabetes complications (retinopathy, end-stage renal disease, stroke, and ischemic heart disease), duration of type 2 diabetes mellitus (new-onset, < 5 years, and ≥ 5 years), number of OHAs administered, or amount of insulin used [28].

On the day of the health examination, anthropometric measurements, including height, weight and WC were measured, and BMI was calculated as follows: weight (kg) divided by square of the height (m^2^). Laboratory tests were performed to assess the serum levels of fasting glucose, total cholesterol, TGs, high density lipoprotein cholesterol, ALT, AST, and GGT. Estimated glomerular filtration rates (eGFR) were calculated from serum creatinine levels using the Modification of Diet in Renal Disease Study Group [29].

### Statistical analysis

Continuous and categorical variables were expressed as mean ± standard deviation and numbers (%). Analysis of variance for continuous variables and chi-square tests for categorical variables were used for evaluating FLI-based differences. For skewed distributed continuous variables, geometric mean values with 95% confidence interval (CIs) were used.

We performed Cox proportional hazards analysis to evaluate the association between the FLI and/or BARD score and mortality and obtain hazard ratios (HRs). Multivariable adjusted models were adjusted for age and sex (model 1); lifestyle habits (smoking status, alcohol consumption, and physical activity), income level, comorbidities (hypertension and dyslipidemia), ALT, CCI score, presence of type 2 diabetes complications, and diabetes duration in addition to age and sex (model 2); Furthermore, stratification analysis was performed according to sex, age (< 40, 40–64, and ≥ 65 years), and BMI in kg/m^2^ (< 18.5 [underweight], 18.5–23 [normal], 23–25 [overweight], and ≥ 25 [obesity]). Statistical analyses were performed using SAS (version 9.4; SAS Institute, Cary, NC, USA). Two-tailed *p*-values < 0.05 were considered statistically significant.

## Results

### Baseline characteristics

Table 1 shows the baseline characteristics of the study population according to FLI score: 40.5%, 33.8%, and 25.7% of the participants were in the < 30, 30–59, ≥ 60 FLI groups, respectively.

**Table 1:** Baseline characteristics according to fatty liver index

	Fatty liver index			*p*-value
	< 30	30–59	≥ 60	
	(n = 813,691) 40.5%	(n = 678,049) 33.8%	(n = 516,035) 25.7%	
Age, years	59.2 ± 12.9	58.8 ± 11.7	54.2 ± 12.1	< 0.001
< 40	55,977 (6.9)	35,861 (5.3)	59,187 (11.5)	< 0.001
40–64	458,800 (56.4)	416,227 (61.4)	347,340 (67.3)	
≥ 65	298,914 (36.7)	225,961 (33.3)	109,508 (21.2)	
Sex				< 0.001
Male	373,049 (45.9)	400,478 (59.1)	372,398 (72.2)	
Female	440,642 (54.2)	277,571 (40.9)	143,637 (27.8)	
Smoking				< .0001
Non	553,647 (68.0)	391,127 (57.7)	236,309 (45.8)	
Former	115,671 (14.2)	128,594 (19.0)	108,157 (21.0)	
Current	144,373 (17.7)	158,328 (23.4)	171,569 (33.3)	
Alcohol drinking				< 0.001
Non	581,309 (71.4)	419,766 (61.9)	245,960 (47.7)	
Mild to moderate	232,382 (28.6)	258,283 (38.1)	270,075 (52.3)	
Regular physical activity	179,016 (22.0)	139,140 (20.5)	91,562 (17.7)	< 0.001
Low income level^a^	177,620 (21.8)	141,907 (20.9)	107,943 (20.9)	< 0.001
Comorbidity				
Hypertension	398,785 (49.0)	404,652(59.7)	328,008 (63.6)	< 0.001
Dyslipidemia	296,241 (36.4)	298,919(44.1)	252,320 (48.9)	< 0.001
CCI score	2.4 ± 2.1	2.3 ± 2.1	2.0 ± 2.0	< 0.001
DM complication				
Retinopathy	96,632 (11.9)	61,667 (9.1)	30,486 (5.9)	< 0.001
ESRD	3,010 (0.4)	1,375 (0.2)	639 (0.1)	< 0.001
Stroke	4,946 (0.6)	4048 (0.6)	2,260 (0.4)	< 0.001
Ischemic heart disease	98,760 (12.1)	88,888 (13.1)	57,305 (11.1)	< 0.001
DM duration				< 0.001
New-onset	278,555 (34.3)	240,331 (35.4)	231,933 (45.0)	
< 5 years	236,684 (29.1)	223,672 (33.0)	170,182 (33.0)	
≥ 5 years	298,052 (36.6)	214,046 (31.6)	113,920 (22.1)	
Number of OHA use				< 0.001
0	309,275 (38.0)	259,072 (38.2)	242,942 (47.1)	
1	375,712 (46.2)	319,144 (47.1)	209,595(40.6)	
≥ 2	128,704 (15.8)	99,833 (14.7)	63,498 (12.3)	
Use of insulin	81,394 (10.0)	54,840 (8.1)	32,534 (6.3)	< 0.001
Body mass index, kg/m^2^	22.7 ± 2.3	25.5 ± 2.3	28.2 ± 3.3	< 0.001
Waist circumference, cm	78.9 ± 6.4	86.6 ± 5.7	93.3 ± 7.4	< 0.001
Systolic BP, mmHg	126.0 ± 15.8	129.8 ± 15.5	132.3 ± 15.6	< 0.001
Diastolic BP, mmHg	76.5 ± 9.9	79.3 ± 9.9	82.0 ± 10.3	< 0.001
Fasting glucose, mg/dL	140.3 ± 46.5	144.4 ± 46.0	151.7 ± 48.1	< 0.001
Total cholesterol, mg/dL	188.4 ± 39.8	198.7 ± 41.6	209.3 ± 45.0	< 0.001
HDL-C, mg/dL	54.4 ± 21.6	50.5 ± 22.1	49.4 ± 27.6	< 0.001
TG, mg/dL^b ^	101.0 (100.9–101.1)	156.4 (156.3–156.6)	230.4 (230.0–230.7)	< 0.001
eGFR, mL/min/1.73m^2^	84.7 ± 35.4	83.5 ± 34.6	85.3 ± 37.9	< 0.001
AST, IU/L ^b^	21.9 (21.9–21.9)	25.0 (25.0–25.0)	31.7 (31.6–31.7)	< 0.001
ALT, IU/L^b^	19.5 (19.5–19.5)	26.1 (26.1–26.1)	37.1 (37.1–37.2)	< 0.001
GGT, IU/L^b^	21.0 (20.9–21.0)	35.4 (35.4–35.55)	65.9 (65.8–66.0)	< 0.001

Values are presented as mean ± standard deviation or median (range) for continuous variables and number (%) for categorical variables

*CCI* charlson comorbidity index, *ESRD* end-stage renal disease, *DM* diabetes mellitus, *OHA* oral glucose agent, *BP* blood pressure, *HDL-C* high density lipoprotein-cholesterol, *TG* triglyceride, *eGFR* estimated glomerular filtration rate, *AST* aspartate transaminase, *ALT* alanine transferase, *GGT* gamma-glutamyl transferase

^a^Lower 20% of income

^b^Geometric mean with 95% confidence interval

The mean age in the FLI ≥ 60 group was lower than that in the other groups (54.2 vs. 58.8 and 59.2 years). The FLI ≥ 60 group predominantly comprised men (72.2%), whereas only 45.9% of the FLI < 30 group were men. People with an FLI ≥ 60 were more likely to be former or current smokers and alcohol consumers than other groups of people. Hypertension and dyslipidemia were more prevalent in the FLI ≥ 60 group. The FLI < 30 group had a higher proportion of patients with a diabetes duration ≥ 5 years, number of OHAs ≥ 2, and insulin use than the other groups. Higher BMI and WC values; systolic/diastolic blood pressure; serum levels of fasting glucose, total cholesterol, TG, ALT, AST and GGT were found in the FLI ≥ 60 group (*P* < 0.001).

### Association between fatty liver index and all-cause/cause-specific mortality

In the total population, 222,242 deaths occurred over a median follow-up period of 8.1 years with a mortality rate of 14.3 per 1000 person-years.

**Fig. 1:**
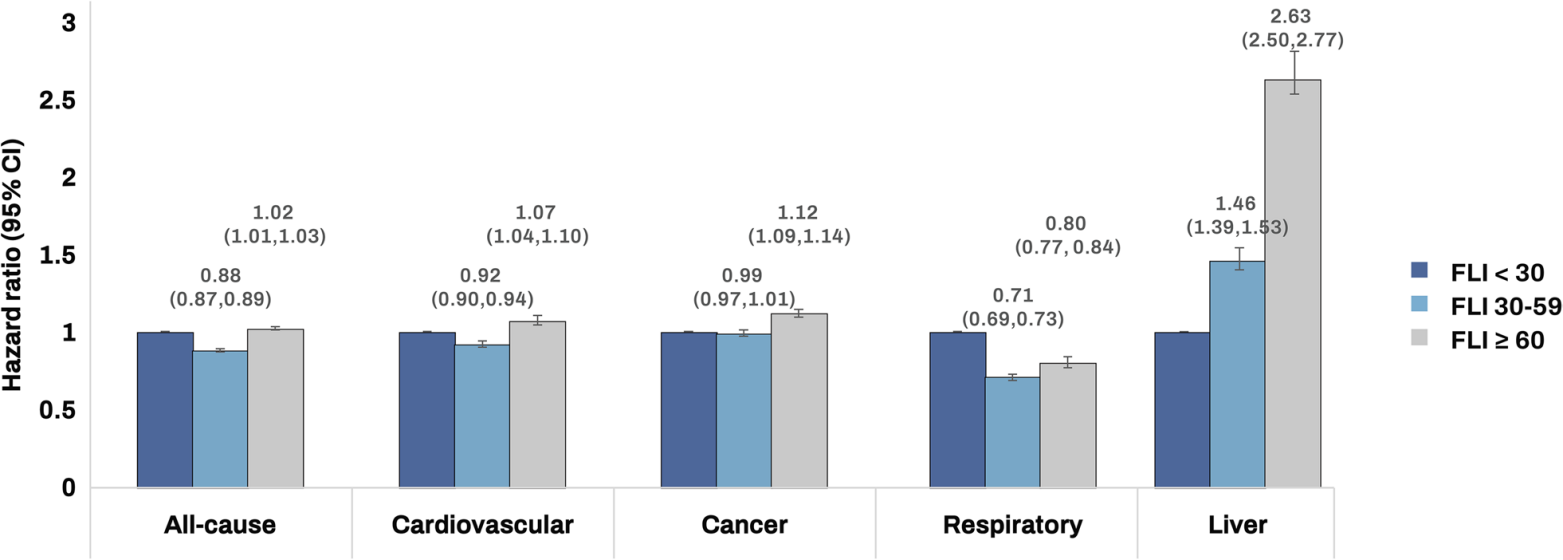
Risk for all-cause, cardiovascular diseases-, cancer-, respiratory disease-related, and liver disease-related mortality according to fatty liver index. *FLI* fatty liver index, *CI* confidence interval

Table 2 shows the different HR trends by FLI category for all-cause and cause-specific mortality. In the multivariate analysis, patients with an FLI ≥ 60 exhibited slightly high risk of all-cause mortality (FLI ≥ 60: HR, 95% CI = 1.02, 1.01–1.03 and FLI 30–59: 0.88, 0.87–0.89) compared to those in the reference group (FLI < 30). The risks of CVD- and cancer-related mortality increased in patients with an FLI ≥ 60 (HR, 95% CI = 1.07, 1.04–1.10, and 1.12, 1.09–1.14, respectively). The risks of liver disease-related mortality linearly increased with higher FLI scores (FLI ≥ 60: HR, 95% CI = 2.63, 2.50–2.77 and FLI 30–59: 1.46, 1.39–1.53, Fig. 1). When we included kidney function as a covariate, consistent results were found Additional file 1: Table S1). The associations of an FLI ≥ 60 with cause-specific mortality were similarly observed in both male and females while the positive association between FLI and all-cause mortality maintained only in female Additional file 1: Table S2).

**Table 2:** All-cause and cause-specific mortality by fatty liver index

Fatty liver index	Number	Death	PYs	Incidence rate (per 1000 PY)	Hazard ratio (95% confidence interval)	
					Model 1	Model 2
All Cause of mortality						
< 30	813,691	103,961	6274880.77	16.57	1(Ref.)	1(Ref.)
30–59	678,049	72,774	5298066.55	13.74	0.87 (0.86,0.88)	0.88 (0.87,0.89)
≥ 60	516,035	45,507	4021847.66	11.31	0.99 (0.98,1.01)	1.02 (1.01,1.03)
CVD-specific mortality						
< 30	813,691	22,307	6274880.77	3.55	1(Ref.)	1(Ref.)
30–59	678,049	15,910	5298066.55	3.00	0.92 (0.90,0.94)	0.92 (0.90,0.94)
≥ 60	516,035	9,351	4021847.66	2.33	1.03 (1.01,1.06)	1.07 (1.04,1.10)
Cancer-specific mortality						
< 30	813,691	27,417	6274880.77	4.37	1(Ref.)	1(Ref.)
30–59	678,049	22,670	5298066.55	4.28	0.99 (0.97,1.01)	0.99 (0.97,1.01)
≥ 60	516,035	15,247	4021847.66	3.79	1.13 (1.11,1.16)	1.12 (1.09,1.14)
Respiratory disease-related mortality						
< 30	813,691	12,210	6274880.77	1.95	1 (Ref.)	1 (Ref.)
30–59	678,049	6,235	5298066.55	1.18	0.67 (0.65, 0.69)	0.71 (0.69, 0.73)
≥ 60	516,035	3,289	4021847.66	0.82	0.71 (0.68, 0.73)	0.80 (0.77, 0.84)
Liver disease-related mortality						
< 30	813,691	2,775	6274880.77	0.44	1(Ref.)	1(Ref.)
30–59	678,049	3,568	5298066.55	0.67	1.47 (1.40,1.55)	1.46 (1.39,1.53)
≥ 60	516,035	4,264	4021847.66	1.06	2.80 (2.66,2.94)	2.63 (2.50,2.77)

Model 1 was adjusted for age and sex; Model 2 was adjusted for smoking status, alcohol consumption, physical activity, low income, alanine aminotransferase, hypertension, dyslipidemia, Charlson comorbidity index, diabetes complication, and diabetes duration and in addition to covariates in model 1

*PY* person year, *CVD* cardiovascular diseases

### Association between the BARD score and all-cause/cause-specific mortality

**Fig. 2:**
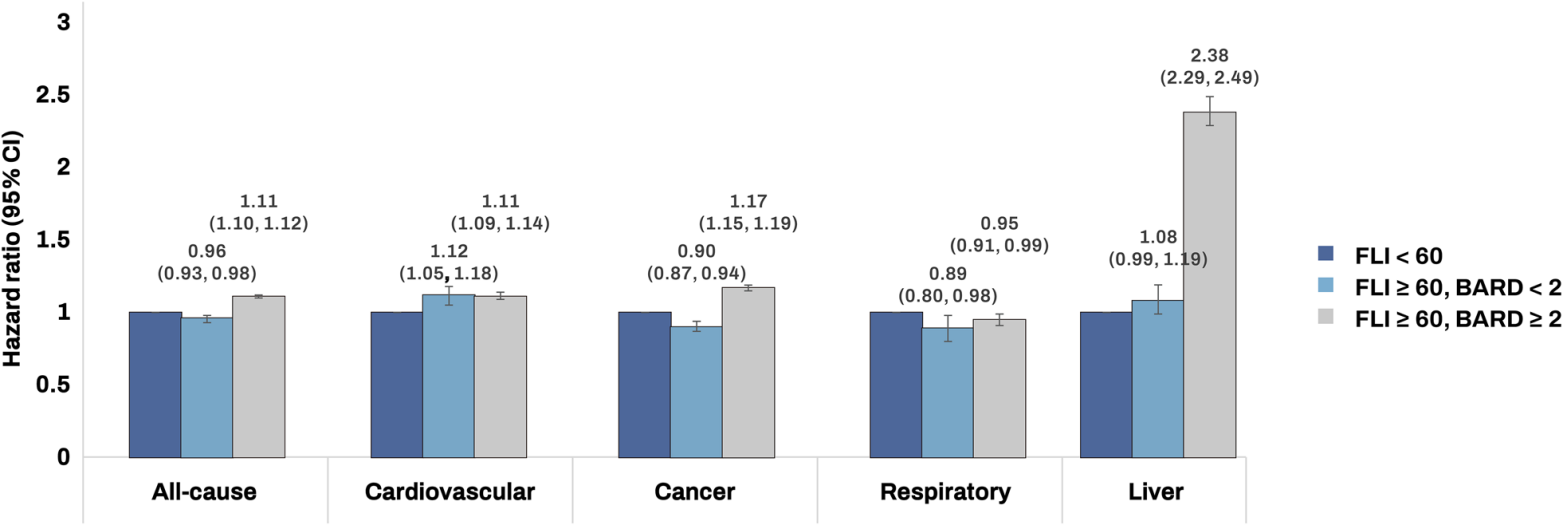
Risk for all-cause, cardiovascular diseases-, cancer-, respiratory disease-related, and liver disease-related mortality according to fatty liver index and BARD score. *FLI* fatty liver index, *CI* confidence interval

Among 516,035 patients with type 2 diabetes mellitus with FLI scores ≥ 60, 404,610 (78.4%) had advanced fibrosis (BARD score ≥ 2). Compared with those with FLI scores < 60, patients with an FLI ≥ 60 and fibrosis (BARD score ≥ 2) exhibited increasing risks of all-cause (HR, 95% CI = 1.11,1.10–1.12), CVD- (HR, 95% CI = 1.11, 1.09–1.14), cancer- (HR, 95% CI = 1.17, 1.15–1.19), and liver disease-related mortality (HR, 95% CI = 2.38, 2.29–2.49), while decreased respiratory disease- related mortality (HR, 95% CI = 0.95, 0.91–0.99) in the multivariate model (Table 3, Fig. 2).

**Table 3:** All-cause and cause-specific mortality according to advanced fibrosis

	N	Death	Duration (PYs)	Incidence rate (per 1000 PY)	Hazard ratio (95% confidence interval)	
					Model 1	Model 2
All Cause of mortality						
FLI < 60	1,491,740	176,735	11572947.32	15.27	1(Ref.)	1(Ref.)
FLI ≥ 60, BARD < 2	111,425	6,664	882056.70	7.56	0.94 (0.91, 0.96)	0.96 (0.93, 0.98)
FLI ≥ 60, BARD ≥ 2	404,610	38,843	3139790.96	12.37	1.08 (1.07, 1.09)	1.11 (1.10, 1.12)
CVD-specific mortality						
FLI < 60	1,491,740	38,217	11572947.32	3.30	1(Ref.)	1(Ref.)
FLI ≥ 60, BARD < 2	111,425	1,345	882056.70	1.52	1.00 (0.95, 1.06)	1.12 (1.05, 1.18)
FLI ≥ 60, BARD ≥ 2	404,610	8,006	3139790.96	2.55	1.08 (1.06, 1.11)	1.11 (1.09, 1.14)
Cancer-specific mortality						
FLI < 60	1,491,740	50,087	11572947.32	4.33	1(Ref.)	1(Ref.)
FLI ≥ 60, BARD < 2	111,425	2,327	882056.70	2.64	0.96 (0.92, 1.00)	0.90 (0.87, 0.94)
FLI ≥ 60, BARD ≥ 2	404,610	12,920	3139790.96	4.11	1.18 (1.16, 1.20)	1.17 (1.15, 1.19)
Respiratory disease-related mortality						
FLI < 60	1,491,740	18,445	11572947.32	1.59	1 (Ref.)	1 (Ref.)
FLI ≥ 60, BARD < 2	111,425	424	882056.70	0.48	0.72 (0.65, 0.79)	0.89 (0.80, 0.98)
FLI ≥ 60, BARD ≥ 2	404,610	2,865	3139790.96	0.91	0.85 (0.81, 0.88)	0.95 (0.91, 0.99)
Liver disease-related mortality						
FLI < 60	1,491,740	6,343	11572947.32	0.55	1(Ref.)	1(Ref.)
FLI ≥ 60, BARD < 2	111,425	484	882056.70	0.55	1.29 (1.18, 1.42)	1.08 (0.99, 1.19)
FLI ≥ 60, BARD ≥ 2	404,610	3,780	3139790.96	1.20	2.51 (2.41, 2.61)	2.38 (2.29, 2.49)

Model 1 was adjusted for age and sex; Model 2 was adjusted for smoking status, alcohol consumption, physical activity, low income, alanine aminotransferase, hypertension, dyslipidemia, Charlson comorbidity index, diabetes complication and diabetes duration in addition to covariates in model 1

*PY* person year, *CVD* cardiovascular diseases, *FLI* fatty liver index

Next, we considered AST/ALT ratio (AAR) alone as surrogate biomarker of fibrosis because all individuals had diabetes (all had 1 point in BARD score). Consistently, patients with an FLI ≥ 60 and fibrosis (AAR ≥ 0.8) showed increasing risks of all-cause, CVD-, cancer-, and liver disease-related mortality compared with those with FLI < 60, Additional file 1: Table S3**)**.

### Stratification analysis by age and BMI

We conducted subgroup analyses stratified by age and BMI to confirm different subgroup associations. On performing stratified analysis by age group, a stronger relationship was noted in middle-aged groups (40–64 years) than in other age groups (FLI ≥ 60: HR, 95% CI = 1.05, 0.94–1.19 for ages < 40; 1.12, 1.10–1.15 for ages 40–64; and 0.98, 0.91–0.99 for ages ≥ 65) (*P* for interaction < 0.001). We stratified the participants according to BMI categories (< 18.5, 18.5–23, 23–25, and ≥ 25), and there was an increased risk of all-cause mortality with FLI score in all BMI groups. A greater association was observed in those with underweight group (BMI < 18.5) than in the other BMI groups (FLI ≥ 60: HR, 95% CI = 2.19, 1.83–2.63 for BMI < 18.5; 1.97, 1.90–2.04 for BMI of 18.5–23; 1.61, 1.57–1.66 for BMI of 23–25; and 1.38, 1.35–1.41 for BMI ≥ 25) (*P* for interaction < 0.001) Additional file 1.

## Discussion

This is, to the best of our knowledge, the first study to investigate the association of all-cause and cause-specific mortality with FLI scores in patients with type 2 diabetes mellitus. All-cause, CVD-, cancer-, and liver disease-related mortality increased with an FLI ≥ 60 in patients with type 2 diabetes mellitus. Moreover, advanced hepatic fibrosis assessed using the BARD score was significantly associated with an increased risk of mortality in patients with type 2 diabetes mellitus. Our findings suggest that determining the presence of hepatic steatosis and/or fibrosis potentially plays a role in risk stratification of mortality outcomes in patients with type 2 diabetes mellitus.

### Association of fatty liver index with mortality

Consistent with our results, previous studies have demonstrated a significant association between all-cause mortality and FLI, and it has not been limited to people with type 2 diabetes mellitus [30, 31]. Regarding cause-specific mortality, including that related to CVD, cancer, and liver diseases, similar trends have been observed in previous studies based on the general population [32]. [26] However, the presence of NAFLD in patients with type 2 diabetes mellitus aggravates the complications of diabetes, rendering it difficult to achieve proper glycemic goals [33, 34]. The coexistence of NAFLD and type 2 diabetes mellitus potentially amplifies the risk of mortality. Although limited evidence exists for the association between NAFLD and mortality or cause-of-death in people with type 2 diabetes mellitus, a previous study reported that NAFLD was associated with increased risks of CVD (HR [95% CI]: 1.70 [1.52–1.90]), hepatocellular carcinoma (HCC) (19.12 [11.71–31.2]), non-HCC cancer (1.10 [0.94–1.29]), and all-cause (1.60 [1.40–1.83]) mortality among people with type 2 diabetes mellitus [8]. In this study, the HR of liver disease-related mortality was the greatest (HR = 2.6) in the FLI ≥ 60 group among cause-specific mortalities. The conflicting results from the two studies may be related to the heterogeneous criteria used to categorize causes of death and diagnostic criteria for NAFLD. There was an inverse association with high FLI and respiratory disease-mortality in this study. Although there are few studies regarding NAFLD and respiratory mortality, Lin et al. reported an inverse relationship between NAFLD and respiratory disease-related mortality, consistent with our findings [35].

### Association of hepatic fibrosis with mortality

In this study, advanced hepatic fibrosis was associated with all-cause, CVD-, cancer-, and liver disease-related mortality. Regarding the increased risk of CVD-related mortality, a recent study consistently revealed that advanced hepatic fibrosis was significantly associated with the risks of CVD events and mortality [36]. Advanced liver fibrosis, measured by hepatic transient elastography, was a risk marker while severe steatosis was a protective factor for cardiovascular complications and mortality in individuals with type 2 diabetes and NAFLD [37]. Collectively, these results suggest that advanced hepatic fibrosis is potentially useful as a screening tool for predicting both hepatic and extrahepatic adverse outcomes in patients with type 2 diabetes mellitus. Accordingly, the appropriate assessment of fibrosis stage is recommended in patients with type 2 diabetes mellitus and NAFLD [13, 38].

### Stratification analysis

On performing a stratification analysis by age, the increased risk of all-cause mortality in the high-FLI group was highest among middle aged patients (40–64 years). We further stratified the participants by BMI, and there was an increased risk of all-cause mortality with high FLI in all BMI groups. In the high FLI group, the increased risk of all-cause death was greatest in the underweight group. These findings indicate that the prognosis of lean NAFLD may be worse in patients with type 2 diabetes mellitus, thus exhibiting consistency with the findings of previous studies that were not limited to people with type 2 diabetes mellitus [39]. Thus, providing intensive NAFLD management may be helpful, especially to middle-aged and lean patients with type 2 diabetes mellitus and NAFLD.

### Liver disease-related mortality

Among the risks of cause-specific mortality in this study, the HR of liver disease-related mortality was the greatest in patients with high FLI scores and/or advanced fibrosis. This finding may be related to the association between hepatic lipid accumulation and an increased risk of type 2 diabetes mellitus as well as adipose tissue and insulin resistance [40]. Recently, the association between obesity and the risk of type 2 diabetes mellitus has been reported to be mediated by the presence of NAFLD [41]. This association may be bidirectional, and the presence of type 2 diabetes mellitus in patients with excessive fatty liver infiltration potentially contributes to an increased risk of all-cause and liver-related mortality [42].

### Clinical implication and limitations

Emerging evidence supports that some antidiabetic agents may improve NAFLD or hepatic fibrosis when added to lifestyle changes in patients with type 2 diabetes mellitus [38]. Thiazolidinedione treatment has been reported to improve the histologic features of hepatic steatosis, inflammation, and ballooning and reduce hepatic fibrosis progression in patients with prediabetes or type 2 diabetes mellitus [43]. Treatment with sodium-glucose cotransporter 2 inhibitors reportedly leads to reduced liver fat content [44] and has been associated with a lower risk of major hepatic events in type 2 diabetes mellitus [45]. Thus, proactive pharmacologic treatment has been recommended in patients with diabetes and concomitant advanced liver disease or in those at high risk of liver disease [46].

In this study, all-cause and cause-specific mortality increased in patients with high FLI and BARD scores, suggesting the prognostic potential of these serum markers in the risk stratification of mortality-related outcomes in patients with type 2 diabetes mellitus. In the absence of data on effective primary screening tools for NAFLD in type 2 diabetes mellitus, identifying high-risk groups and providing interventions, including lifestyle changes and medication, may be helpful.

Notwithstanding, this study has several limitations. First, imaging studies or pathology are mandatory for the diagnosis of hepatic steatosis; however, these methods are expensive and generally not feasible for screening fatty liver in a large population-based cohort. Although FLI cannot distinguish simple steatosis from steatohepatitis and fibrosis, FLI was used to define NAFLD in several previous large-population studies using claims data [47, 48]. Nevertheless, the biomarkers for detecting hepatic steatosis are not as accurate in patients with diabetes as they are in the general population since these markers were not developed for the population with diabetes or only included a minority of patients with type 2 diabetes mellitus. In addition, some of these biomarkers rely on the diabetic status and impaired blood glucose levels, thus not allowing for the accurate estimation of the prevalence of NAFLD [49]. In addition, the BARD score, which was used as a surrogate marker of advanced fibrosis in this study, is less specific than other biomarkers, such as the NAFLD fibrosis or fibrosis-4 scores. Because the Korean NHIS database does not include information regarding platelet count or albumin level, we could not assess other liver-fibrosis prediction scores. Second, since this was an observational study, unmeasured variables, such as insulin resistance, family history of type 2 diabetes mellitus, or hemoglobin A1C levels, could have influenced the results; thus, we could not ascertain the causality of the associations. However, we attempted to thoroughly adjust for possible confounding factors. Third, since mortality data were obtained through linkage to the NHIS, deaths that occurred outside Korea were not captured, and the possibility of a potential misclassification of some causes of death cannot be excluded. Fourth, since antidiabetic therapy employing sodium glucose co transporter 2 inhibitors was initiated in 2013, the conclusions of the study could be outdated. Finally, the study population consisted of Korean subjects; therefore, the results of this study cannot be generalized to other ethnic groups. More research is needed to validate our results and elucidate the mechanisms underlying our findings.

In conclusion, hepatic steatosis and/or advanced fibrosis, as assessed using the FLI and BARD scores, were significantly associated with the risks of overall and cause-specific mortality in patients with type 2 diabetes mellitus.

## Supplementary Information

**Additional file 1 Table S1. **All-cause and cause-specific mortality by fatty liver index with additional adjustment for kidney function. **Table S2.** Hazard ratios (95% confidence interval) for all-cause mortality and cause-specific mortality according to fatty liver index by sex. **Table S3.** All-cause and cause-specific mortality according to advanced fibrosis using aspartate aminotransferase/alanine transaminase ratio. **Table S4.** Stratified analysis: hazard ratios (95% confidence interval) for all-cause mortality according to fatty liver index by age groups and body mass index.

## Data Availability

We used the claim data provided by the Korean National Health Insurance Service (NHIS) database. Data can only be accessed by visiting the NHIS datacenter, after approval from data access committee of NHIS (https://nhiss.nhis.or.kr/bd/ab/bdaba001cv.do).
