# Supplementary material for: The association of fatty liver index and BARD score with all-cause and cause-specific mortality in patients with type 2 diabetes mellitus: a nationwide population-based study
Source: Cardiovasc Diabetol. 2022 Dec 6;21:273. doi: 10.1186/s12933-022-01691-6 (PMC9727979; doi:10.1186/s12933-022-01691-6)
Supplement: Supplementary file 1 — Additional file 1 Table S1. All-cause and cause-specific mortality by fatty liver index with additional adjustment for kidney function. Table S2. Hazard ratios (95% confidence interval) for all-cause mortality and cause-specific mortality according to fatty liver index by sex. Table S3. All-cause and cause-specific mortality according to advanced fibrosis using aspartate aminotransferase/alanine transaminase ratio. Table S4. Stratified analysis: hazard ratios (95% confidence interval) for all-cause mortality according to fatty liver index by age groups and body mass index. [file 12933_2022_1691_MOESM1_ESM.docx]

**Table S1. All-cause and cause-specific mortality by fatty liver index with additional adjustment for kidney function**

| Fatty liver index | Number | Death | PYs | Incidence rate  (per 1,000 PY) | Hazard ratio (95% confidence interval) | | |
| --- | --- | --- | --- | --- | --- | --- | --- |
|  |  |  |  |  | Model 1 | Model 2 |  |
| All Cause of mortality | |  |  |  |  |  |  |
| < 30 | 813,691 | 103,961 | 6274880.77 | 16.57 | 1 (Ref.) | 1 (Ref.) |  |
| 30-59 | 678,049 | 72,774 | 5298066.55 | 13.74 | 0.87 (0.86, 0.88) | 0.87 (0.86, 0.88) |  |
| ≥ 60 | 516035 | 45,507 | 4021847.66 | 11.31 | 1.00 (0.99, 1.01) | 0.99 (0.98, 1.00) |  |
| CVD-specific mortality | |  |  |  |  |  |  |
| < 30 | 813,691 | 22,307 | 6274880.77 | 3.55 | 1 (Ref.) | 1 (Ref.) |  |
| 30-59 | 678,049 | 15,910 | 5298066.55 | 3.00 | 0.91 (0.89, 0.93) | 0.90 (0.88, 0.92) |  |
| ≥ 60 | 516,035 | 9,351 | 4021847.66 | 2.33 | 1.04 (1.01, 1.07) | 1.03 (1.00, 1.06) |  |
| Cancer-specific mortality | |  |  |  |  |  |  |
| < 30 | 813,691 | 27,417 | 6274880.77 | 4.37 | 1 (Ref.) | 1 (Ref.) |  |
| 30-59 | 678,049 | 22,670 | 5298066.55 | 4.28 | 0.99 (0.97, 1.01) | 0.99 (0.97, 1.01) |  |
| ≥ 60 | 516035 | 15,247 | 4021847.66 | 3.79 | 1.12 (1.10, 1.14) | 1.12 (1.09, 1.14) |  |
| Respiratory disease-related mortality | | |  |  |  |  |  |
| < 30 | 813,691 | 12,210 | 6274880.77 | 1.95 | 1 (Ref.) | 1 (Ref.) |  |
| 30-59 | 678,049 | 6,235 | 5298066.55 | 1.18 | 0.71 (0.68, 0.73) | 0.70 (0.68, 0.72) |  |
| ≥ 60 | 516,035 | 3,289 | 4021847.66 | 0.82 | 0.80 (0.76, 0.83) | 0.79 (0.75, 0.82) |  |
| Liver disease-related mortality | | |  |  |  |  |  |
| < 30 | 813,691 | 2,775 | 6274880.77 | 0.44 | 1(Ref.) | 1 (Ref.) |  |
| 30-59 | 678,049 | 3,568 | 5298066.55 | 0.67 | 1.46 (1.39, 1.54) | 1.46 (1.39, 1.54) |  |
| ≥ 60 | 516,035 | 4,264 | 4021847.66 | 1.06 | 2.64 (2.51, 2.78) | 2.64 (2.51, 2.78) |  |

PY, person year; CVD, cardiovascular diseases

Model 1 was adjusted for age, sex, smoking status, alcohol consumption, physical activity, low income, alanine aminotransferase, hypertension, dyslipidemia, Charlson comorbidity index, diabetes complication, diabetes duration and estimated glomerular filtration rate (eGFR); Model 2 was adjusted for age, sex, smoking status, alcohol consumption, physical activity, low income, alanine aminotransferase, hypertension, dyslipidemia, Charlson comorbidity index, diabetes complication, diabetes duration and chronic kidney disease (eGFR <60 ml/min/1.73m^2^).

**Table S2. Hazard ratios (95% confidence interval) for all-cause mortality and cause-specific mortality according to fatty liver index by sex**

| FLI | Male | | | | | Female | | | | |
| --- | --- | --- | --- | --- | --- | --- | --- | --- | --- | --- |
|  | N | Event | Duration | IR, per 1000 PY | HR (95% CI) | N | Event | Duration | IR, per 1000 PY | HR (95% CI) |
| All Cause of mortality | | | |  |  |  |  |  |  |  |
| < 30 | 373,049 | 57,126 | 2827316.62 | 20.21 | 1 (Ref.) | 440,642 | 46,835 | 3447564.15 | 13.58 | 1 (Ref.) |
| 30-59 | 400,478 | 45,082 | 3102262.23 | 14.53 | 0.86 (0.85, 0.88) | 277,571 | 27,692 | 2195804.33 | 12.61 | 0.89 (0.88, 0.91) |
| ≥ 60 | 372,398 | 31,435 | 2894059.72 | 10.86 | 0.96 (0.95, 0.98) | 143,637 | 14,072 | 1127787.95 | 12.47 | 1.09 (1.07, 1.11) |
| CVD-specific mortality | | | |  |  |  |  |  |  |  |
| < 30 | 373,049 | 10,919 | 2827316.62 | 3.86 | 1 (Ref.) | 440,642 | 11,388 | 3447564.15 | 3.30 | 1 (Ref.) |
| 30-59 | 400,478 | 8,965 | 3102262.23 | 2.89 | 0.91 (0.88, 0.94) | 277,571 | 6,945 | 2195804.33 | 3.16 | 0.92 (0.90, 0.95) |
| ≥ 60 | 372,398 | 5,976 | 2894059.72 | 2.06 | 1.01 (0.98, 1.05) | 143,637 | 3,375 | 1127787.95 | 3.99 | 1.10 (1.06, 1.15) |
| Cancer-specific mortality | | | |  |  |  |  |  |  |  |
| < 30 | 373,049 | 16,956 | 2827316.62 | 5.99 | 1 (Ref.) | 440,642 | 10,461 | 3447564.15 | 3.03 | 1 (Ref.) |
| 30-59 | 400,478 | 15,427 | 3102262.23 | 4.97 | 0.98 (0.96, 1.00) | 277,571 | 7,243 | 2195804.33 | 3.30 | 1.02 (0.99, 1.05) |
| ≥ 60 | 372,398 | 11,316 | 2894059.72 | 3.91 | 1.10 (1.07, 1.13) | 143,637 | 3,931 | 1127787.95 | 3.49 | 1.22 (1.17, 1.26) |
| Respiratory disease-related mortality | | | |  |  |  |  |  |  |  |
| < 30 | 373,049 | 7,406 | 2827316.62 | 2.62 | 1 (Ref.) | 440,642 | 4,804 | 3447564.15 | 1.39 | 1 (Ref.) |
| 30-59 | 400,478 | 4,074 | 3102262.23 | 1.31 | 0.69 (0.66, 0.72) | 277,571 | 2,161 | 2195804.33 | 0.98 | 0.73 (0.70, 0.77) |
| ≥ 60 | 372,398 | 2,231 | 2894059.72 | 0.77 | 0.73 (0.69, 0.77) | 143,637 | 1,058 | 1127787.95 | 0.94 | 0.94 (0.88, 1.01) |
| Liver disease-specific mortality | | | |  |  |  |  |  |  |  |
| < 30 | 373,049 | 1,873 | 2827316.62 | 0.66 | 1 (Ref.) | 440,642 | 902 | 3447564.15 | 0.26 | 1 (Ref.) |
| 30-59 | 400,478 | 2,650 | 3102262.23 | 0.85 | 1.42 (1.34, 1.51) | 277,571 | 918 | 2195804.33 | 0.42 | 1.52 (1.39, 1.67) |
| ≥ 60 | 372,398 | 3,428 | 2894059.72 | 1.18 | 2.48 (2.33, 2.63) | 143,637 | 836 | 1127787.95 | 0.74 | 3.04 (2.76, 3.35) |

IR, incidence rate; PY, person year; HR, hazard ratio; CI, confidence interval; FLI, fatty liver index; CVD, cardiovascular diseases

Hazard ratio was adjusted for age, sex, smoking status, alcohol consumption, physical activity, low income, alanine aminotransferase, diabetes mellitus, hypertension, dyslipidemia, Charlson comorbidity index, diabetes complication and diabetes duration.

**Table S3.** **All-cause and cause-specific mortality according to advanced fibrosis using aspartate aminotransferase/alanine transaminase ratio**

|  |  | Death | Duration (PYs) | Incidence rate  (per 1,000 PY) | Hazard ratio (95% confidence interval) | | |
| --- | --- | --- | --- | --- | --- | --- | --- |
|  | N |  |  |  | Model 1 | Model 2 | Model 3 |
| All Cause of mortality | | | |  |  |  |  |
| FLI < 60 | 1,491,740 | 176,735 | 11572947.32 | 15.27 | 1 (Ref.) | 1(Ref.) | 1(Ref.) |
| FLI ≥ 60, AAR<0.8 | 225,897 | 11,426 | 1784661.43 | 6.40 | 0.90 (0.88, 0.92) | 0.91 (0.90, 0.93) | 0.91 (0.89, 0.93) |
| FLI ≥ 60, AAR ≥ 0.8 | 290,138 | 34,081 | 2237186.23 | 15.24 | 1.12 (1.11, 1.13) | 1.15 (1.14, 1.16) | 1.14 (1.13, 1.15) |
| CVD-specific mortality | | | |  |  |  |  |
| FLI < 60 | 1,491,740 | 38,217 | 11572947.32 | 3.30 | 1 (Ref.) | 1 (Ref.) | 1 (Ref.) |
| FLI ≥ 60, AAR<0.8 | 225,897 | 2,313 | 1784661.43 | 1.30 | 0.96 (0.92, 1.00) | 0.93 (0.90, 0.98) | 0.93 (0.89, 0.97) |
| FLI ≥ 60, AAR ≥ 0.8 | 290,138 | 7,038 | 2237186.23 | 3.15 | 1.11 (1.08, 1.14) | 1.10 (1.08, 1.13) | 1.09 (1.06, 1.11) |
| Cancer-specific mortality | | | |  |  |  |  |
| FLI < 60 | 1,491,740 | 50,087 | 11572947.32 | 4.33 | 1 (Ref.) | 1(Ref.) | 1(Ref.) |
| FLI ≥ 60, AAR<0.8 | 225,897 | 4,096 | 1784661.43 | 2.30 | 0.95 (0.92, 0.98) | 0.96 (0.93, 1.00) | 0.97 (0.93, 1.00) |
| FLI ≥ 60, AAR ≥ 0.8 | 290,138 | 11,151 | 2237186.23 | 4.98 | 1.23 (1.20, 1.25) | 1.25 (1.22, 1.27) | 1.25 (1.22, 1.28) |
| Respiratory disease-related mortality | | | |  |  |  |  |
| FLI < 60 | 1,491,740 | 18,445 | 11572947.32 | 1.59 | 1 (Ref.) | 1 (Ref.) | 1 (Ref.) |
| FLI ≥ 60, AAR<0.8 | 225,897 | 680 | 1784661.43 | 0.38 | 0.67 (0.62, 0.72) | 0.69 (0.64, 0.75) | 0.69 (0.64, 0.75) |
| FLI ≥ 60, AAR ≥ 0.8 | 290,138 | 2,609 | 2237186.23 | 1.17 | 0.88 (0.85, 0.92) | 0.94 (0.90, 0.98) | 0.94 (0.90, 0.98) |
| Liver disease-related mortality | | | |  |  |  |  |
| FLI < 60 | 1,491,740 | 6,343 | 11572947.32 | 0.55 | 1 (Ref.) | 1(Ref.) | 1(Ref.) |
| FLI ≥ 60, AAR<0.8 | 225,897 | 762 | 1784661.43 | 0.43 | 1.13 (1.05, 1.22) | 1.21 (1.12, 1.31) | 1.21 (1.12, 1.31) |
| FLI ≥ 60, AAR ≥ 0.8 | 290,138 | 3,502 | 2237186.23 | 1.57 | 2.85 (2.73, 2.97) | 2.94 (2.81, 3.07) | 2.95 (2.82, 3.08) |

PY, person year; CVD, cardiovascular diseases; FLI, fatty liver index; AAR, aspartate aminotransferase/alanine transaminase ratio

Model 1 was adjusted for age and sex; Model 2 was adjusted for smoking status, alcohol consumption, physical activity, low income, hypertension, dyslipidemia, Charlson comorbidity index, diabetes complication and diabetes duration in addition to covariates in model 1; Model 3 was adjusted for estimated glomerular filtration rate in addition to covariates in model 2.

**Table S4. Stratified analysis: hazard ratios (95% confidence interval) for all-cause mortality according to fatty liver index by age groups and body mass index.**

|  | FLI | N | Event | Duration | IR, per 1000 PY | HR (95% CI) | p for interaction |
| --- | --- | --- | --- | --- | --- | --- | --- |
| Age groups (years) | |  |  |  |  |  |  |
| <40 | < 30 | 55,977 | 470 | 447002.99 | 1.05 | 1(Ref.) | <0.001 |
|  | 30-59 | 35,861 | 334 | 286454.89 | 1.17 | 0.85 (0.74,0.98) |  |
|  | ≥ 60 | 59,187 | 704 | 468992.96 | 1.50 | 1.05 (0.94,1.19) |  |
| 40-64 | < 30 | 458,800 | 21,797 | 3663297.32 | 5.95 | 1(Ref.) |  |
|  | 30-59 | 416,227 | 19,487 | 3321067.66 | 5.87 | 0.92 (0.90,0.94) |  |
|  | ≥ 60 | 347,340 | 17,582 | 2739889.15 | 6.42 | 1.12 (1.10,1.15) |  |
| ≥65 | < 30 | 298,914 | 81,694 | 2164580.47 | 37.74 | 1(Ref.) |  |
|  | 30-59 | 225,961 | 52,953 | 1690544 | 31.32 | 0.87 (0.86,0.88) |  |
|  | ≥ 60 | 109,508 | 27,221 | 812965.55 | 33.48 | 0.98 (0.91,0.99) |  |
| BMI (kg/m^2^) |  |  |  |  |  |  |  |
| <18.5 | < 30 | 30,869 | 95,71 | 207095.13 | 46.21 | 1(Ref.) | <0.001 |
|  | 30-59 | 1,187 | 505 | 7391.41 | 68.32 | 1.87 (1.71,2.04) |  |
|  | ≥ 60 | 284 | 119 | 1831.4 | 64.98 | 2.19 (1.83,2.63) |  |
| 18.5-23 | < 30 | 404,993 | 59,262 | 3089661.32 | 19.18 | 1(Ref.) |  |
|  | 30-59 | 84,924 | 14,216 | 640859.3 | 22.18 | 1.23 (1.21,1.26) |  |
|  | ≥ 60 | 16,697 | 3,315 | 122618.26 | 27.04 | 1.97 (1.90,2.04) |  |
| 23-25 | < 30 | 244,881 | 24,574 | 1922807.51 | 12.78 | 1(Ref.) |  |
|  | 30-59 | 199,744 | 22,706 | 1556695.86 | 14.59 | 1.16 (1.14,1.18) |  |
|  | ≥ 60 | 56,456 | 6,457 | 435837.05 | 14.82 | 1.61 (1.57,1.66) |  |
| ≥25 | < 30 | 132,948 | 10,554 | 1055316.82 | 10.00 | 1(Ref.) |  |
|  | 30-59 | 392,194 | 35,347 | 3093119.98 | 11.43 | 1.10 (1.07,1.12) |  |
|  | ≥ 60 | 442,598 | 35,616 | 3461560.95 | 10.29 | 1.38 (1.35,1.41) |  |

IR, incidence rate; PY, person year; HR, hazard ratio; CI, confidence interval; FLI, fatty liver index; BMI, body mass index

Hazard ratio was adjusted for age, sex, smoking status, alcohol consumption, physical activity, low income, alanine aminotransferase, diabetes mellitus, hypertension, dyslipidemia, Charlson comorbidity index, diabetes complication and diabetes duration.
